# The efficacy and safety of ginger (*Zingiber officinale*) rhizome extract in outpatients with COVID-19: A randomized double-blind placebo-control clinical trial

**DOI:** 10.1097/MD.0000000000038289

**Published:** 2024-05-31

**Authors:** Ali Ameri, Mehdi Farashahinejad, Parivash Davoodian, Omid Safa, Mehdi Hassaniazad, Mohsen Parsaii, Behnoosh Heidari, Soheil Hassanipour, Boshra Akhlaghi, Mohammad Fathalipour

**Affiliations:** a Student Research Committee, Faculty of Pharmacy, Hormozgan University of Medical Sciences, Bandar Abbas, Iran; b Infectious and Tropical Diseases Research Center, Hormozgan Health Institute, Hormozgan University of Medical Sciences, Bandar Abbas, Iran; c Department of Clinical Pharmacy, Faculty of Pharmacy, Hormozgan University of Medical Sciences, Bandar Abbas, Iran; d Gastrointestinal and Liver Diseases Research Center, Guilan University of Medical Sciences, Rasht, Iran; e Endocrinology and Metabolic Research Center, Hormozgan University of Medical Sciences, Bandar Abbas, Iran; f Department of Pharmacology and Toxicology, Faculty of Pharmacy, Hormozgan University of Medical Sciences, Bandar Abbas, Iran.

**Keywords:** Complementary medicine, COVID-19, Ginger, Pharmacotherapy, Randomized controlled trial

## Abstract

**Background:**

Ginger, a potent antiviral, anti-inflammatory, and antioxidant remedy, is a potential therapeutic option for COVID-19. However, there was not enough clinical evidence about ginger and COVID-19. We evaluated the efficacy and safety of ginger on clinical and paraclinical features in outpatients with COVID-19.

**Methods:**

In this randomized controlled trial, the outpatients with confirmed COVID-19 were randomly assigned in a 1:1 ratio to receive ginger (1000 mg 3 times a day for 7 days) or placebo. The primary outcome was viral clearance after the end of the intervention. Oxygen saturation (S_P_O_2_), body temperature, respiratory rate (RR), hospital admission, and the incidence of adverse events were also assessed.

**Results:**

A total of 84 patients (42 in the ginger and 42 in the control groups) were randomized. The viral clearance was not statistically improved in the ginger group (41.6%) compared to the placebo group (42.8%). The findings indicated that S_P_O_2_, body temperature, and RR had no significant difference between the groups at the end of the intervention. The imaging finding indicated pulmonary infiltrate significantly reduced on the 7^th^ day of the intervention in the ginger group. The percentage of patients with S_P_O_2_ <96% in the ginger group decreased over the study compared to the placebo group. Moreover, the need for hospital admission and the incidence of adverse drug events were not different between the groups over the follow-up period.

**Conclusions:**

Ginger had no significant impact on the clinical and paraclinical parameters of patients. However, this intervention demonstrated a safe profile of adverse events and reduced pulmonary infiltrate.

**Trial registration:**

The trial was registered as IRCT20200506047323N1.

## 1. Introduction

Severe acute respiratory syndrome coronavirus 2 (SARS-CoV-2) is a newly emerged virus from the coronaviridae family. This virus has caused coronavirus disease 2019 (COVID-19). Due to the high speed of the spread of SARS-CoV-2, COVID-19 caused a global pandemic and spread rapidly worldwide.^[1]^ The pathogenesis of COVID-19 includes various pathways, including oxidative stress, hyper-inflammatory responses, cytokine storm, lung vascular leakage, and extensive apoptosis and necrosis of lung cells.^[2,3]^

This disease has several consequences, in particular, lung inflammation^[4]^ and fibrosis,^[5]^ severe hypoxia,^[6]^ cardiac arrhythmia,^[7]^ acute kidney injury,^[8]^ neurological disorders,^[9]^ and liver dysfunction.^[10]^ These consequences result in a high rate of morbidity and mortality.^[11]^ A number of vaccines and medicines have been approved or authorized against COVID-19. However, we still face a lot of outpatients with COVID-19 due to the *emergence* of new variants of SARS-CoV-2.^[12]^ Hence, large efforts are ongoing to find efficient treatments to control and improve the disease.

Computational studies indicated phytocompounds of ginger (*Zingiber officinale* Roscoe, Zingiberacae) might have a beneficial affinity to spike protein of SARS-CoV-2.^[13]^ Also, in vitro study was shown bioactive agents in ginger roots such as xanthorrhizo could inhibit the replication of SARS-CoV-2 and other viruses of the coronaviridae family.^[14]^

Ginger has several active ingredients with antioxidant, anti-inflammatory, and immunomodulatory activities.^[15]^ During the viral infection, ginger protects the viral-infected cells from reactive oxygen species by antioxidant effects. Moreover, the immunomodulatory effects of ginger reduce inflammation.^[16]^

Previous studies have shown ginger had beneficial effects of ginger on patients infected with the hepatitis C virus.^[17]^ Ginger could also prevent attachment and internalization of the human respiratory syncytial virus to human respiratory tract cell lines and stimulation of interferon-beta secretion. Furthermore, ginger has an anti-inflammatory impact by preventing the production of prostaglandins and inflammatory cytokines.^[18]^ This medicinal plant indicated an antiviral effect against the chikungunya virus.^[19]^

Clinical studies have demonstrated herbal compounds containing ginger and Echinacea have beneficial effects on clinical symptoms of COVID-19 such as dyspnea, muscle pain, and cough, and reduce the rate of hospitalization.^[20]^ Clinical studies on moderate COVID-19 patients showed ginger-containing compounds could reduce recovery time and duration of illness. These compounds improved clinical symptoms, including fever, cough, and gastrointestinal upsets.^[21,22]^ A clinical study conducted in China showed patients with moderate COVID-19 who received ginger supplements had shorter length of stay (LOS) in hospital significantly.^[23]^

Considering the fact that ginger has potential antiviral, antioxidant, and anti-inflammatory effects and the need for a highly efficient pharmacological agent for the management of COVID-19, we evaluated the efficacy and safety of ginger on clinical and paraclinical features in outpatients with confirmed COVID-19.

## 2. Methods

### 2.1. Study design

This study was a randomized, double-blind, placebo-controlled clinical trial to assess the efficacy and safety of ginger in outpatients infected with SARS-CoV-2. The trial was conducted at the Shahid Mohammadi Hospital, Bandar Abbas, Iran. The study was also undertaken in accordance with the guidelines of the Declaration of Helsinki and the principles of the International Conference on Harmonization Good Clinical Practice. Written Informed consent was obtained from all patients and participants had the right to leave the study whenever they wanted. The Ethics Committee of Hormozgan University of Medical Sciences approved the trial (IR.HUMS.REC.1399.130). The study was registered with the Iranian Registry of Clinical Trials (IRCT20200506047323N1).

### 2.2. Participation

Patients were recruited between July 1, 2022 and December 31, 2022. Both men and women with an age of ≥ 18 years, confirmed diagnosis of SARS-CoV-2 based on the positive real-time polymerase chain reaction (RT-PCR) test, referred to the Respiratory Outpatients Department were eligible to enroll in the study.

The patients with peripheral capillary oxygen saturation (S_P_O_2_) < 94% who needed to be admitted to the hospital as well as the underlying disorders, including uncontrolled cardiovascular diseases, severe renal and liver failures, and uncontrolled thyroid diseases were considered as exclusion criteria. All patients with a history of known allergy to ginger, current use of warfarin, selective serotonin reuptake inhibitors, monoamine oxidase inhibitors, diuretics, and antiarrhythmic, and pregnant or breastfeeding women were also excluded. If complications associated with ginger were observed during the study, the patient will be excluded from the study and supportive measures will be taken.

### 2.3. Randomization and blinding

Eligible patients were randomly assigned to either ginger or control groups in a 1:1 ratio using an interactive web-based system. Stratified block randomization was used with a block size of 6 to create the randomization sequence. Sealed envelopes were used to protect the randomization sequence. A special code was allocated to every patient to conceal their identity, and patients were assigned to groups based on their unique code. All patients, clinicians, nurses, research coordinators, and investigators were blinded to the group assignment.

### 2.4. Intervention

Patients in the ginger group received the standard regimen for COVID-19 based on the Iranian Ministry of Health and Medical Education treatment protocol plus ginger-based herbal tablets (Vomigone, Dineh Iran Pharmaceutical Company, IRC: 9406633051781240) at a dose of 1000 mg (2 tablets) 3 times a day for a period of 7 days. Patients in the placebo group received the standard regimen plus Vomigone-like placebo tablets (Dineh Iran Pharmaceutical Company, Iran) at a dose of 2 tablets 3 times a day for a period of 7 days. The standard treatment regimen for outpatient COVID-19 based on the Iranian Ministry of Health and Medical Education protocol included hydroxychloroquine sulfate (Amin Chemical and Pharmaceutical Company, Isfahan) at a dose of 200 mg twice a day for a period of 7 days. Taking the medicines was assessed daily by telephone follow-up.

### 2.5. Outcomes measures

The primary endpoint of this trial was the viral clearance of SARS-CoV-2 in the nasopharyngeal samples assessed by RT-PCR after 7 days of randomization. The peripheral capillary oxygen saturation (S_P_O_2_), body temperature, and respiratory rate (RR) were compared between the study groups at the end of the intervention as secondary outcomes. The improvement in paraclinical parameters including, hematological, biochemical, liver function, and infection-related indices was also evaluated. All patients were followed up for one more week and any hospital admission were recorded. The incidence of adverse events was documented during the intervention.

### 2.6. Statistical analysis

The study sample size was calculated upon the assumption that the clinical improvement in outpatients with COVID-19 by day 7 would be 95% in the treatment group and 55% in the control group, according to previous studies.^[24–26]^ Considering a power of 80% and a significance level of 0.05, this study needed 35 participants in each arm. Accounting for a probable 20% dropout rate, 42 patients were required in each group.

The variables were presented as the mean (standard deviation) or frequency (percentages) of patients in each category. The Fisher exact test was used for the comparison of groups regarding categorical variables and the independent *t* test for between-group comparison of continuous variables (normality checked with Kolmogorov Spiridonov test). The analysis of covariance (ANCOVA) analysis was employed to compare mean differences of paraclinical variables measured at the baseline and at the end of the intervention for the study groups.

The efficacy outcomes were assessed in the per-protocol population who had received complete treatment regimens. The safety outcome was studied in the intent-to-treat population who had received at least one dose of the medications. The SPSS version 18.0 (SPSS Inc., Chicago, IL) was used for statistical analysis, and *P* < .05 was considered statistically significant.

## 3. Results

### 3.1. Study population

**Figure 1.**
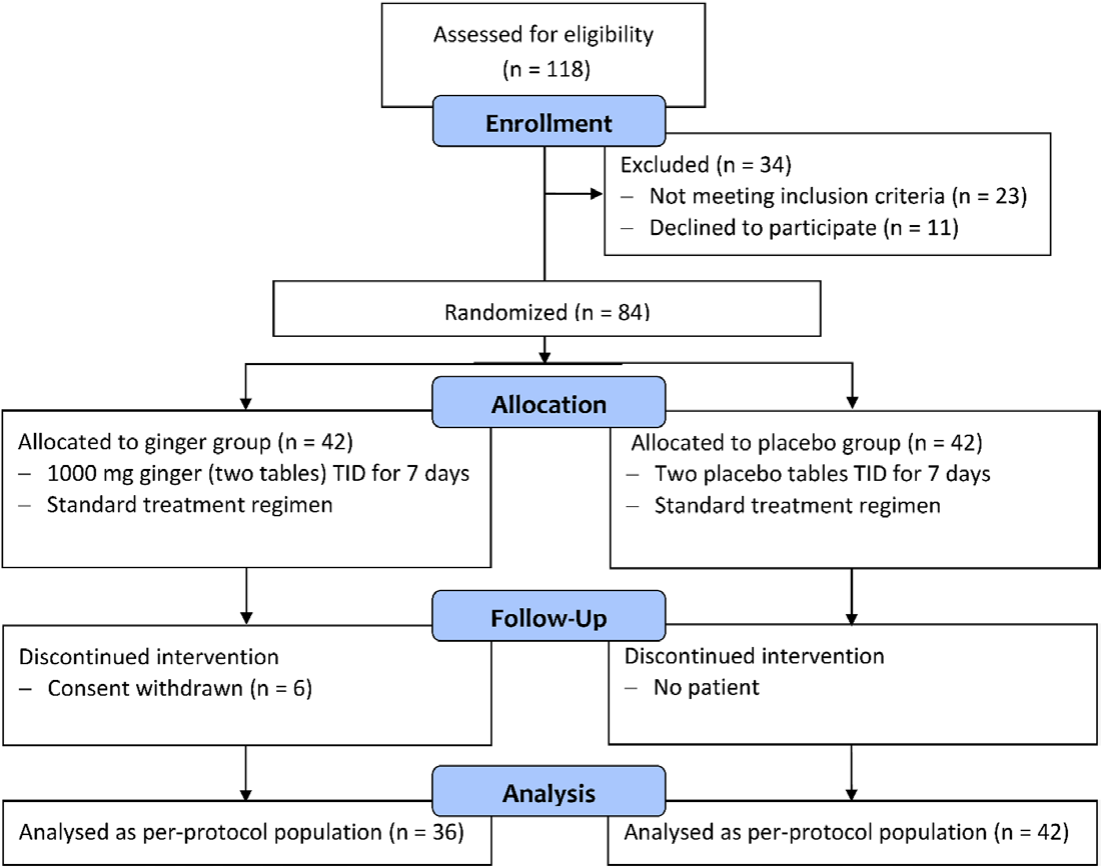
Overview of the patient enrollment and treatment assignment.

A total of 118 patients with laboratory-confirmed SARS-CoV-2 infection were recruited, and after assessment of eligibility criteria, 84 patients were randomly allocated to the ginger (n = 42) and placebo (n = 42) groups. Six patients in the ginger group were excluded from the study after randomization due to withdrawal of consent, not because of the incidence of any adverse events, and the remaining 78 patients completed the treatment regimen and were defined as the per-protocol population (Fig. 1).

The mean age of patients was 35.46 (SD, 11.90) years, and 51 patients (65.4%) were men in the per-protocol population. A total of 8 patients (10.3%) had an S_P_O_2_ of < 96%, 28 patients (35.9%) had a body temperature of ≥ 37.5°C, and 10 patients (12.8%) had a RR of ≥ 16/min at the baseline. Baseline demographic criteria were not statistically different between the ginger and placebo groups except the number of patients with S_P_O_2_ < 96% in the ginger group was significantly higher than in the placebo group (Table 1). Baseline laboratory and imaging findings were similar between the studied groups (Table 2).

**Table 1:** Baseline demographic and clinical characteristics of the patients.

Characteristics	Ginger group (n = 36)	Placebo group (n = 42)	*P* value
Age, yr	36.38 ± 13.19	34.57 ± 10.62	.517
Sex, male	20 (55.6)	31 (73.8)	.102
Exposure history	9 (25)	18 (47.3)	.098
Symptoms onset until intervention	3.50 ± 1.03	3.55 ± 1.09	.844
Comorbidities			
Diabetes	1 (2.8)	1 (2.4)	.912
Hypertension	4 (11.1)	1 (2.4)	.117
Cardiovascular disease	1 (2.8)	2 (4.8)	.650
Obesity	1 (2.8)	1 (2.4)	.894
Chronic lung disease	2 (5.6)	1 (2.4)	.632
Thyroid disorders	0 (0.0)	1 (2.4)	.351
Clinical features			
S_P_O_2_ < 96%	7 (19.4)	1 (2.6)	.021
Temperature ≥ 37.5°C	10 (27.8)	18 (42.9)	.237
RR ≥ 16/min	5 (13.9)	5 (11.9)	.528

Values were expressed as mean ± SD or n (%).

Comparison between groups was performed using the independent *t* test or Fisher exact test.

RR = respiratory rate, S_P_O_2_ = peripheral capillary oxygen saturation, Temperature = body temperature.

**Table 2:** Baseline laboratory and imaging findings of the participants.

Characteristics		Ginger group (n = 36)	Placebo group (n = 42)	*P* value
Hematologic, ×10^9^/L				
Hematocrit	Baseline	41.01 ± 3.10	40.30 ± 5.98	.505
	Day 7	41.34 ± 3.40	40.83 ± 3.83	.533
WBC	Baseline	7.00 ± 2.35	7.41 ± 2.39	.445
	Day 7	7.04 ± 2.41	7.35 ± 2.48	.574
Neutrophil	Baseline	76.00 ± 15.93	72.74 ± 12.25	.310
	Day 7	77.03 ± 11.15	74.64 ± 11.37	.352
Lymphocytes	Baseline	17.01 ± 9.30	20.77 ± 11.88	.122
	Day 7	15.83 ± 9.13	19.45 ± 11.04	.117
Platelets	Baseline	168.54 ± 28.24	168.69 ± 36.10	.985
	Day 7	163.58 ± 36.33	160.75 ± 35.02	.727
Biochemical				
Sodium, mEq/L	Baseline	140.58 ± 3.28	140.80 ± 2.46	.757
	Day 7	140.47 ± 2.38	140.88 ± 2.19	.433
Potassium, mEq/L	Baseline	4.66 ± 2.50	4.17 ± 0.33	.217
	Day 7	4.25 ± 0.38	4.15 ± 0.27	.216
FBS, mg/dL	Baseline	89.92 ± 9.78	86.74 ± 7.76	.114
	Day 7	87.92 ± 7.83	86.33 ± 5.67	.306
Hemoglobin, g/L	Baseline	13.71 ± 1.11	13.75 ± 1.43	.901
	Day 7	13.78 ± 10	13.75 ± 1.20	.912
BNU, mg/dL	Baseline	21.56 ± 9.43	20.40 ± 6.24	.522
	Day 7	21.31 ± 9.50	20.62 ± 5.48	.692
Creatinine, mg/dL	Baseline	0.83 ± 0.22	0.77 ± 0.15	.200
	Day 7	0.84 ± 0.23	0.79 ± 0.16	.256
ALT, units/L	Baseline	36.19 ± 1.00	39.86 ± 23.60	.389
	Day 7	37.00 ± 9.92	35.02 ± 8.75	.353
AST, units/L	Baseline	27.61 ± 7.95	25.10 ± 5.04	.095
	Day 7	27.22 ± 8.46	24.90 ± 5.36	.147
LDH, units/L	Baseline	218.72 ± 51.73	241.74 ± 101.61	.223
	Day 7	213.64 ± 49.52	217.19 ± 19.25	.669
CRP, mg/dL	Baseline	10.00 ± 11.50	8.60 ± 8.27	.537
	Day 7	8.97 ± 5.71	7.17 ± 4.57	.132
Ferritin, ng/mL	Baseline	147.31 ± 67.12	173.55 ± 144.98	.322
	Day 7	143.22 ± 66.33	140.60 ± 39.84	.830
Coagulation function				
D-dimer, mg/L	Baseline	220.57 ± 90.61	202.21 ± 78.30	.340
	Day 7	226.50 ± 90.06	203.02 ± 57.72	.184
Imaging				
Ground-glass pattern	Baseline	23 (63.9)	21 (50.0)	.257
	Day 7	7 (19.4)	18 (42.9)	.031
Consolidation	Baseline	8 (22.2)	14 (33.3)	.321
	Day 7	2 (5.6)	11 (26.2)	.017

Values were expressed as mean ± SD, or n (%).

Comparison between groups was performed using the *t* test, or the Fisher exact test.

ALT = alanine aminotransferase, AST = aspartate aminotransferase, BUN = blood nitrogen urea, CRP = C-reactive protein, LDH = lactate dehydrogenase, PT = prothrombin time, TIBC = total iron binding capacity, WBC = white blood cells.

### 3.2. Efficacy

The viral clearance in the nasopharyngeal samples on day 7 was not statistically improved in the ginger group (41.6%) compared to the placebo group (42.8%). The results demonstrated S_P_O_2_ (*P* = .182), temperature (*P* = .705), and RR (*P* = .121) had no significant difference between the groups at the end of the intervention. The rate of hospital admission in the ginger group (2.8%) was not statistically different compared to the placebo group (0.0%) over the follow-up period (*P* = .462). Considering the baseline data, the number of patients with worse prognosis (only S_P_O_2_ < 96%, not temperature ≥ 37.5°C and RR ≥ 16/min) at the end of treatment was significantly decreased in the ginger group compared to the placebo groups. The clinical outcomes are presented in Table 3.

**Table 3:** Clinical outcomes of the patients with confirmed COVID-19 after treatment.

Outcomes	Ginger group (n = 36)	Placebo group (n = 42)	*P* value
Primary outcomes			
SARS-CoV-2 clearance	15 (41.6)	18 (42.8)	.916
Secondary outcomes			
S_P_O2	98.19 ± 1.09	98.45 ± 0.55	.182
<96%	2 (5.6)	0 (0.0)	.210
Temperature	36.90 ± 0.30	36.88 ± 0.25	.705
≥37.5°C	2 (5.6)	0 (0.0)	.210
Respiratory rate	13.66 ± 1.98	13.09 ± 1.18	.121
≥16/min	2 (5.6)	1 (2.4)	.442
Hospital admission	1 (2.8)	0 (0.0)	.462

Values were expressed as mean (SD) or n (%).

Comparison between groups was performed using the independent *t* test or Fisher exact test.

Data were collected after 7 days of randomization except for hospital admission (documented within 14 days of randomization). The outcomes were assessed in the per-protocol population who had received complete treatment regimens.

RR = respiratory rate, S_P_O_2_ = peripheral capillary oxygen saturation, Temperature = body temperature.

**Figure 2.**
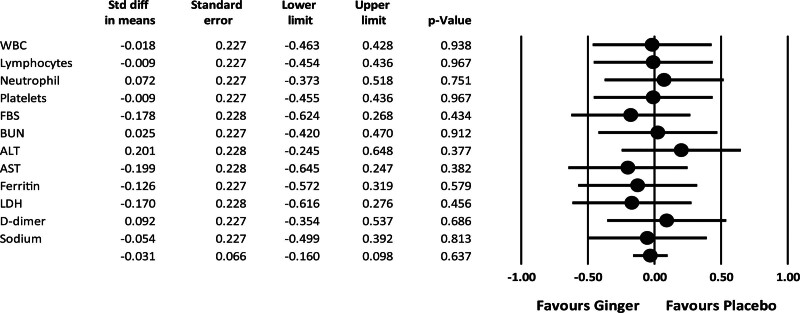
The change in the mean difference of paraclinical variables was measured at the baseline and at the end of intervention for the study groups.

The comparison of laboratory findings between ginger and placebo groups at baseline and the seventh day of intervention demonstrated no significant difference (Table 2). Imaging findings show that on the seventh day of the intervention, the rate of pulmonary infiltrate in the ginger group was significantly lower than in the placebo group (Table 2). The comparison of laboratory findings between baseline and the seventh day of intervention in patients of each group demonstrated no significant difference, except blood neutrophil counts in the placebo group (*P* = .034) (Table 4). The clinical symptoms from the baseline and the seventh day of intervention had no significant differences between the ginger and placebo groups except sore throat on day 7 (*P* = .020), abdominal pain at baseline (*P* = .011), nausea at baseline (*P* = .007), anorexia at baseline (*P* = .019) (Table 5). ANCOVA analyses also found no significant differences in the mean differences of paraclinical parameters between the ginger and the placebo groups (Fig. 2).

**Table 4:** Laboratory characteristics of the patients before and 7 days of intervention.

	Ginger group (n = 36)						Placebo Group (n = 42)					
	Baseline		Day 7		Mean difference	*P* value	Baseline		Day 7		Mean difference	*P* value
	Mean	SD	Mean	SD			Mean	SD	Mean	SD		
WBC, ×10^9^/L	6.69	2.20	6.85	2.40	−0.154	.336	7.32	2.48	7.42	2.53	−0.106	.242
Lymph, ×10^9^/L	16.72	9.60	16.0	9.40	0.725	.076	20.81	11.87	19.95	11.07	0.865	.065
Neut, ×10^9^/L	76.84	16.54	77.0	11.67	−0.158	.960	72.60	12.23	74.09	11.36	−1.48	.034
PLT, ×10^9^/L	166.45	27.80	162.50	37.12	3.95	.562	166.92	33.28	162.52	35.71	4.39	.477
FBS, mg/dL	90.0	10.03	87.90	7.96	2.09	.084	86.27	6.97	86.10	5.44	0.175	.875
BUN, mg/dL	21.68	9.95	21.75	9.86	−0.062	.862	20.25	5.89	20.60	5.32	−0.350	.512
ALT, units/L	36.62	10.21	37.06	10.33	−0.437	.390	37.85	18.21	34.70	8.83	3.15	.231
AST, units/L	27.81	8.29	27.53	8.90	0.281	.883	30.12	32.33	24.77	5.37	5.35	.303
Ferritin, ng/mL	148.12	69.87	145.31	69.23	2.81	.228	158.87	110.80	142.27	40.09	16.40	.341
LDH, units/L	215.61	53.94	213.45	53.31	2.16	.320	232.47	80.77	216.77	19.61	15.70	.190
D-dimer, ng/mL	225.99	94.42	231.93	93.55	−5.94	.085	208.25	75.94	203.66	59.80	4.85	.491
Sodium, mEq/L	140.43	3.38	140.33	2.49	0.100	.831	140.9	2.40	141.0	2.21	0.012	.995

Values were expressed as mean (SD).

Comparison between baseline and Day 7 was performed using the independent *t* test in each group.

ALT = alanine aminotransferase, AST = aspartate aminotransferase, BUN = blood nitrogen urea, FBS = fasting blood glucose, LDH = lactate dehydrogenase, Lymph = lymphocytes, Neut = neutrophils, PLT = platelets, WBC = white blood cells.

**Table 5:** Clinical symptoms of the participants.

Characteristics		Ginger group (n = 36)	Placebo group (n = 42)	*P* value
Fever	Baseline	28 (77.8)	33 (78.6)	.574
	D 7	36 (100)	42 (100)	-
Chills	Baseline	22 (61.1)	27 (64.3)	.478
	D 7	36 (100)	42 (100)	-
Headache	Baseline	29 (80.6)	29 (69.0)	.184
	D 7	1 (2.8)	1 (2.4)	.713
Sore throat	Baseline	25 (69.4)	27 (64.3)	.406
	D 7	0 (0.0)	9 (21.4)	.020
Diarrhea	Baseline	22 (61.1)	30 (71.4)	.235
	D 7	36 (100)	42 (100)	-
Cough	Baseline	24 (66.7)	24 (57.1)	.265
	D 7	8 (22.2)	9 (21.4)	.574
Dyspnea	Baseline	6 (16.7)	7 (16.7)	.617
	D 7	36 (100)	42 (100)	-
Sputum	Baseline	15 (41.7)	10 (23.8)	.075
	D 7	3 (8.3)	4 (9.5)	.587
Rhinorrhea	Baseline	9 (25)	5 (11.9)	.114
	D 7	36 (100)	42 (100)	-
Lethargy	Baseline	30 (83.3)	37 (88.1)	.390
	D 7	8 (22.2)	16 (38.1)	.102
Muscular pain	Baseline	36 (100)	42 (100)	-
	D 7	5 (13.9)	8 (19.0)	.383
Tiredness	Baseline	32 (88.9)	40 (95.2)	.267
	D 7	7 (19.4)	10 (23.8)	.426
Muscle spasm	Baseline	18 (50.0)	29 (69.0)	.690
	D 7	3 (8.3)	1 (2.4)	.252
Chest pain	Baseline	14 (38.9)	15 (35.7)	.478
	D 7	36 (100)	42 (100)	-
Abdominal pain	Baseline	12 (33.3)	26 (61.9)	.011
	D 7	3 (8.3)	4 (9.5)	.587
Nausea	Baseline	20 (55.6)	35 (83.3)	.007
	D 7	36 (100)	42 (100)	-
Anorexia	Baseline	25 (69.4)	38 (90.5)	.019
	D 7	14 (38.9)	11 (26.2)	.170
Smell loss	Baseline	22 (61.1)	31 (73.8)	.170
	D 7	22 (61.1)	32 (76.2)	.117
Taste loss	Baseline	21 (58.3)	23 (54.8)	.465
	D 7	22 (61.1)	23 (54.8)	.369

Values were expressed as n (%).

Comparison between groups was performed using Fisher exact test.

*P < *.05 was considered as statistically significant.

### 3.3. Safety

Overall, more adverse events were reported in the ginger group (6 patients) compared to the placebo group (one patient). Gastrointestinal events were the most prevalent side effects. However, there was no significant difference between the ginger and placebo groups regarding the incidence of adverse drug events (Table 6).

**Table 6:** Adverse drug events of the patient.

Adverse drug events	Ginger group (n = 36)	Placebo group (n = 42)	*P* value
Diarrhea	1 (2.8)	1 (2.4)	.912
Intestinal cramps	1 (2.8)	0 (0.0)	.462
Severe nausea	0 (0.0)	0 (0.0)	-
Severe anorexia	2 (5.6)	0 (0.0)	.210
Heartburn	1 (2.8)	0 (0.0)	.462
Edema	0 (0.0)	0 (0.0)	-
Pruritus	0 (0.0)	0 (0.0)	-
Rash	0 (0.0)	0 (0.0)	-
Hematuria	0 (0.0)	0 (0.0)	-
Lymphadenopathy	0 (0.0)	0 (0.0)	-
Headache	1 (2.8)	0 (0.0)	.462

Values were expressed as n (%).

Comparison between groups was performed using Fisher exact test.

Data were collected within 7 d of randomization. The events were studied in the intent-to-treat population who had received at least one dose of the medications.

## 4. Discussion

A complicated network of oxidative-, immune-, inflammatory, and virus-mediated reactions is involved in the pathogenesis of COVID-19.^[11,12]^ Considering the potential antioxidant, anti-inflammatory, and antiviral effects of ginger,^[27,28]^ we designed the clinical trial, aiming to inhibit the viral life cycle and prevent lung cell inflammation. This was a double-blind, placebo-controlled, randomized trial, where 84 outpatients with COVID-19 were enrolled. The baseline characteristics of the studied groups were generally similar except for the number of patients with S_P_O_2_ < 96%. Regarding the trial findings, there was no significant difference between the ginger and the placebo groups neither in the primary outcome (viral clearance) nor the secondary outcome (temperature, RR, hospital admission, and adverse drug events). Intervention with ginger could improve the number of patients with S_P_O_2_ < 96%. This may be due to the significant reduction of pulmonary infiltrate on the 7th day of the intervention in the ginger group.

Ginger as traditional medicine was used widely for treating various disorders for thousands of years.^[16]^ Several studies have reported ginger can exert inhibitory effects on the virus attachment, entry, replication, and assembling, perhaps via interacting with viral key proteins and enzymes.^[18,29–31]^ Molecular docking studies indicated that ginger active components especially 8-gingerol, 10-gingerol, and 6-gingerol potently exhibit a high binding affinity with several proteins of SARS-CoV-2.^[32–34]^ Ginger is also assumed to affect key fundamental processes involving inflammation and oxidative stress.^[35,36]^

The results from a Bangladeshi study demonstrated consumption of remedies containing ginger cured a few cases of COVID-19 patients.^[37]^ In a study from Saudi Arabia, the hospitalization rate in COVID-19 patients was lower among ginger users compared to nonusers.^[38]^ A clinical trial conducted in Iran showed that combination therapy with ginger and Echinacea in COVID-19 patients diminished some clinical symptoms including, cough, breath shortness, and muscular pain compared to the standard antiviral regimen. The hospitalization rate in the treatment group (2.0%) was also lower than in the control group (6.0%).^[39]^ Some other studies claimed remedies containing ginger would be beneficial for the management of COVID-19.^[40,41]^ In another clinical trial, the Enriched-ginger diet had beneficial impacts on patients with acute respiratory distress syndrome (ARDS) and improved their oxygen saturation, serum inflammatory parameters, and time of mechanical ventilation compared to the control group. However, organ failure and mortality rates were not decreased.^[30]^ Moreover, intervention with ginger could exert a positive effect on pulmonary complications including, fibrosis, ARDS, pneumonia, and sepsis, all of which are the consequence of COVID-19.^[42]^ A clinical study conducted in China in 2022 showed that hospital LOS was significantly shorter than control groups in moderate COVID-19 patients who received oral ginger supplements at a dose of 1.5 g BID until hospital discharge.^[23]^

Overall, the aforementioned evidence demonstrated that ginger might be effective in the management of COVID-19. Although our study shows adding ginger to the standard treatment of outpatients COVID-19 could improve the number of patients with S_P_O_2_ < 96%, this may be due to the significant reduction of pulmonary infiltrate in the ginger group. But, our findings do not support this hypothesis strongly. Because we did not see any significant difference in viral clearance, hospitalization, and laboratory signs. It is necessary, therefore, to discuss the causes of this controversy. First, we have to speculate whether the dosage and the duration proposed to inhibit the viral life cycle and diminish inflammation in these patients were the right ones. Concerning the posology, determination of the exact dose and duration would be infeasible and time-consuming. Hence, we chose a maximum safe dose and duration based on the other clinical trials. Although we did not obtain positive effects, we did show the safety of ginger in this dose and duration.^[43,44]^ Second, the time and stage of disease when intervention with ginger was started. We know that earlier ginger prescriptions could be more effective; however, we know that most of these outpatients would recover spontaneously and would not refer exactly to the start of the symptoms.^[45]^ Finally, we could not conclude the fact that our primary hypothesis was wrong; that is, the disease pathogenesis neither relates to only hyperinflammation nor only oxidative stress. It is indicated that there is a complex pathophysiology in each stage of the disease.

The main limitation of the trial was the small number of enrolled patients. This study was initially protocoled as a preliminary stage; however, given the negative results, further studies were aborted.

## 5. Conclusion

Although the consumption of ginger was safe and well-tolerated, we found ginger had no beneficial impact on the clinical and paraclinical parameters of patients at the studied dose and duration. However, confirmation of the results of this preparatory trial requires more detailed multiple-center trials of COVID-19 with a larger sample size. The combination of ginger and approved medications could be a promising candidate in future trials for the management of COVID-19.

## Acknowledgments

We appreciably thank the trial patients and their families, whose help and participation made this study possible. We would also like to thank the assistance of Dineh Iran Pharmaceutical Company, for preparing the placebo tablets. The Dineh Iran Pharmaceutical Company played no part in the design of the trial the intervention procedures, collection, evaluation, and analysis of data.

## Author contributions

**Conceptualization:** Ali Ameri, Mehdi Farashahinejad, Parivash Davoodian.

**Data curation:** Ali Ameri, Omid Safa, Mehdi Hassaniazad, Mohammad Fathalipour.

**Investigation:** Ali Ameri, Mohsen Parsaii, Behnoosh Heidari, Boshra Akhlaghi, Mohammad Fathalipour.

**Methodology:** Ali Ameri, Mehdi Farashahinejad, Parivash Davoodian.

**Software:** Ali Ameri, Mehdi Farashahinejad, Parivash Davoodian, Soheil Hassanipour.

**Supervision:** Ali Ameri.

**Validation:** Soheil Hassanipour.

**Visualization:** Ali Ameri, Mohsen Parsaii, Behnoosh Heidari, Boshra Akhlaghi, Mohammad Fathalipour.

**Writing – original draft:** Ali Ameri, Omid Safa, Mehdi Hassaniazad, Mohammad Fathalipour.

**Writing – review & editing:** Mohammad Fathalipour.
